# The complete mitochondrial genome of *Tachycines* (*Gymnaeta*) *zorzini* (Orthoptera: Rhaphidophoridae)

**DOI:** 10.1080/23802359.2021.1901624

**Published:** 2021-03-24

**Authors:** Yeying Wang, Haofeng Zhan, Xiaoyan Lv, Binqiang Li, Xiaofei Yang

**Affiliations:** a Key Laboratory of State Forestry Administration on Biodiversity Conservation in Karst Mountainous Area of Southwestern of China, School of Life Sciences, Guizhou Normal University, Guiyang, PR China; b College of Tea Science, Guizhou University, Guiyang, PR China

**Keywords:** *Tachycines* (*Gymnaeta*) *zorzini*, mitochondrial genome, phylogeny

## Abstract

In this study, we elucidated the complete mitochondrial genome (mitogenome) of *Tachycines* (*Gymnaeta*) *zorzini* (accession number MW322826). The circular mitogenome is 15,369-bp-long, including 13 protein-coding genes (PCGs), 22 *tRNA* genes, two *rRNA* genes, and a non-coding control region. The overall base composition is as follows: A, 42.16%; T, 31.75%; C, 15.97%; G, and 10.12%; a slight A + T bias of 73.91%. Phylogenetic analysis of some species of Ensifera revealed that *Tachycines* (*Gymnaeta*) *zorzini* was closer to *Tachycines* (*Tachycines*) *minor*, *Tachycines*, and *Diestrammena* are monophyletic.

*Tachycines* (*Gymnaeta*) *zorzini*, tribe Aemodogryllini of the subfamily Aemodogryllinae, is widely distributed in many karst caves of southern Guizhou, China. In 1992, The genus *Tachycines* is incorporated into *Diestrammena as a subgenus*, so *T.* (*G.*) *zorzini*’s earliest name was *Diestrammena* (*Gymnaeta*) *zorzini* (Rampini et al. 2008; Zhang and Liu 2009). Until 2018, this genus was reinstated Kany’s (1934) system again, treated it as a genus (Qin et al. 2018). Complete mitochondrial genome sequences had been used to construct the phylogenetic tree of Orthoptera (Song et al. 2015) and Ensifera (Zhou et al. 2010, 2014, 2017), but lack of the Aemodogryllinae taxa. Therefore, the complete mitochondrial genome of *T. (G.) zorzini* and its phylogenetic relationships within Aemodogryllinae and other closely taxa, such as Anabropsinae, Prophalangopsidae, and Meconematinae were investigated in this study. The results of this study may contribute to confirm the phylogenetic relationship between *Tachycines* and *Diestrammena*.

The specimen was collected from Shuiniu Cave in Bijie City, Guizhou Province, China (27°12′27.56′′N, 106°4′5.95′′E). The voucher specimens of the species (accession number: SND 20170716) and its DNA (accession number: L34-SND) were stored at School of Life Sciences, Guizhou Normal University, China. The high molecular mitochondrial DNA was extracted from the leg muscle tissue and sequenced using next-generation sequencing. A total of 1.2 G HQ reads in fasta format were obtained and subjected to mitochondrial genome assembly. The complete mitochondrial genome was annotated with MITOS (http://mitos.bioinf.uni-leipzig.de/) (Bernt et al. 2013) with a total length of 15,369 bp, was submitted to the NCBI database under the accession number MW322826, consisting of 13 protein-coding genes (PCGs), 22 *tRNA* genes, two *rRNA* genes, and a control region (D-loop). The overall base composition of the mitogenome is the following: A, 42.16%; T, 31.75%; C, 15.97%; G, and 10.12%; a slight A + T bias of 73.91%. Gene order is highly conserved in comparison to previously described complete genome of another Aemodogryllinae. Phylogenetic tree of the relationships among 11 species of Ensifera insects and one species of Caelifera (*Sinopodisma lushiensis*) in GenBank as outgroup (MN_083189.1) were presented using their available mitogenomes by Bayesian inference methods (Figure 1) using MrBayes version 3.2.1 with a general time reversible (GTR) model of DNA substitution and a gamma distribution rate variation across sites (Ronquist and Huelsenbeck 2003). Phylogenetic analysis (Figure 1) confirms the genus *Tachycines* and *Diestrammena* are monophyletic. *Tachycines* (*Tachycines*) *zorzini* was observed closer to *Tachycines* (*Tachycines*) *minor* formed a monophyletic group, proposed a sister relationship with *Diestrammena* species.

**Figure 1.**
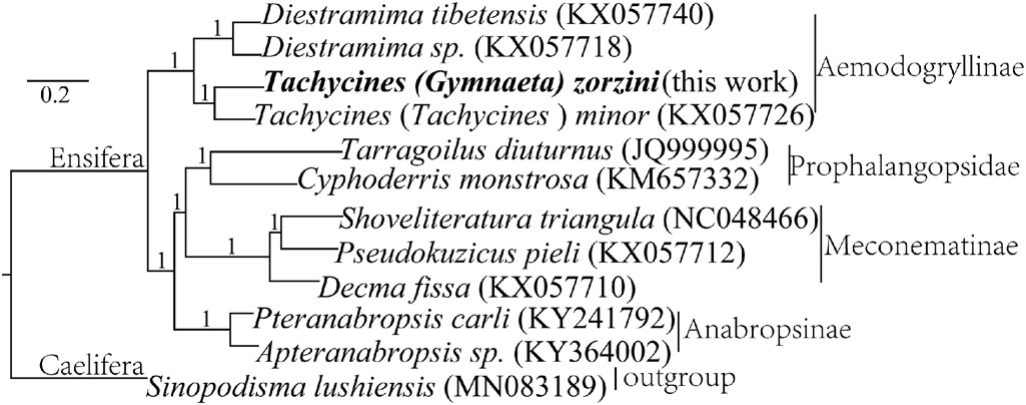
A Bayesian tree of the phylogenetic position of *Tachycines* (*Gymnaeta*) *zorzini* among other Ensifera species. GenBank accession numbers of mitogenome sequences used are shown in parentheses. Branches received Bayesian posterior probabilities (BPP).

## Data Availability

The data that support the findings of this study are openly available in GenBank of NCBI at https://www.ncbi.nlm.nih.gov, reference number MW322826.
